# A Safe and Effective Internal Medicine Procedure Rotation for Medical Students

**DOI:** 10.1007/s40670-025-02510-9

**Published:** 2025-11-28

**Authors:** Jessica A. Blank, Elizabeth Anderson, Elise N. Brannen, Joseph G. Nugent, André M. Mansoor

**Affiliations:** 1 Division of Pulmonary and Critical Care Medicine, Department of Medicine, Northwestern University Feinberg School of Medicine, Chicago, IL USA; 2 Division of Rheumatology, Department of Medicine, Oregon Health & Science University and Portland VA Medical Center, Portland, OR USA; 3 Internal Medicine Residency Program, Department of Medicine, Oregon Health & Science University, Portland, OR USA; 4 Department of Neurosurgery, Oregon Health & Science University, Portland, OR USA; 5 Division of Hospital Medicine, Department of Medicine, Oregon Health & Science University, 3181 SW Sam Jackson Park Rd, Portland, OR 97239 USA

**Keywords:** Medical students, Procedural skills, Bedside procedures, Experiential learning, Internal medicine, Clinical education

## Abstract

**Problem:**

Although procedural rotations in internal medicine residency programs have been shown to increase trainee satisfaction and procedural experience, limited data exist on the implementation and outcomes of similar curricula for medical students.

**Approach:**

Grounded in experiential learning theory, we developed and implemented a multi-week elective procedure rotation for medical students. The curriculum included an online component and hands-on procedural training supervised by experienced faculty. Students completed pre- and post-rotation assessments, including a cognitive assessment and a Likert-style survey measuring procedural volume, confidence, and satisfaction. Paired t-tests and Wilcoxon matched-pairs ranked-sum tests were used to analyze changes in continuous and ordinal outcomes, respectively.

**Outcomes:**

Before the rotation, students reported limited procedural exposure. During the elective, each student performed an average of 18 procedures—most commonly paracentesis—with no major complications. Cognitive assessment scores significantly improved from a pre-rotation average of 66.3% to 87.2% post-rotation (p < 0.001). Statistically significant increases were observed in both procedural confidence and satisfaction with procedural training.

**Next Steps:**

This elective procedure rotation was successfully integrated into the curriculum at a large academic medical center and resulted in substantial gains in student procedural exposure, knowledge, and confidence. These findings underscore the value of experiential learning for medical students and suggest that similar elective rotations at other institutions could enhance procedural training in undergraduate medical education.

## Problem

Knowledge and skills pertaining to bedside procedures are a core competency in multiple surgical and non-surgical specialties. For example, the American Board of Internal Medicine (ABIM) has outlined procedural-based competency requirements for graduates of Internal Medicine residency programs, which include cognitive components (e.g., understanding procedural indications, contraindications, and complications) and technical components (i.e., the act of performing procedures) [1]. Mounting literature describes improvements in the procedural skills of internal medicine residents following the implementation of procedural curricula and standardized rotations. Outcomes have primarily focused on cognitive and technical skills, comfort performing procedures, and satisfaction with educational opportunities for procedures [2–8].

Affording medical students training in common bedside procedures provides experiential learning and may increase the number of supervised procedures they perform before independent practice. Although some literature exists on simulation-based procedural training for medical students, few studies have examined their inclusion in resident procedural rotations or the creation of a dedicated procedural rotation for students [9, 10]. A 2022 systematic review of internal medicine procedure services at US academic medical centers found that learners were exclusively residents [11]. To our knowledge, the feasibility and safety of involving medical students as primary operators on a procedure service have not been reported. Because medical students function in a fundamentally different role than residents—as undifferentiated learners within the hospital—it is unclear whether the approaches and outcomes described in the resident procedure training literature can be directly applied to them.

We introduced an internal medicine procedure rotation into the School of Medicine curriculum at a large academic medical center with the aim of standardizing procedural training and providing interested medical students with procedural experience. We hypothesized that this rotation would be feasible, safe, and would increase procedural opportunities for students while enhancing their understanding of the cognitive aspects of procedures, confidence in performing procedures, and satisfaction with procedural training. Demonstrable growth in these areas, while maintaining patient safety, would support the inclusion of medical students in existing internal medicine procedure rotations at academic medical centers.

## Approach

### Setting

This study was approved by the Institutional Review Board in May 2021 and was carried out in a 576-bed metropolitan academic center. At this institution, there are approximately 150 medical students per class. The undergraduate medical education curriculum includes 18 months of pre-clinical didactics, broken into organ system-based blocks. During the pre-clinical months, students are engaged in weekly clinical preceptorships in various environments, including ambulatory and inpatient. Cadaver-based anatomy labs are held during pre-clinical months along with procedural simulation training opportunities. Students then transition to 30 months of clinical rotations, including seven core rotations and multiple opportunities for electives. Medical students were invited to participate in the study as part of an elective procedure rotation during their clinical years, where they joined an existing procedure service that was designed for internal medicine residents and implemented in 2013 [8]. In the pre-existing rotation, the team consisted of a faculty supervisor and a resident (who was concurrently on call as a back-up resident for other hospital services). At the request of any inpatient service (e.g. General Surgery), the Procedure Service was available to perform paracentesis, thoracentesis, lumbar puncture, and knee arthrocentesis, amounting to a volume of 382 procedures in the 365-day period preceding the involvement of medical students.

Following establishment of the intervention—the medical student elective—medical students in their clinical years of undergraduate medical education could join the Procedure Service as part of an elective rotation, lasting 2 to 8 weeks, depending on student preferences and schedules. The elective was available to medical students between May 2021 and May 2024. Once the service was consulted, students partnered with a resident or faculty member to obtain the patient’s written informed consent, gather equipment, perform the procedure under attending supervision, and document the procedure.

### Curricular Design

As adult learners, medical students are well served by curricula that implement experiential learning, a framework within adult learning theory put forth by Kolb [12]. This is the idea that we learn best when actively performing skills within a safe environment, coached by experienced teachers, and engaged in a cycle of experience, reflection, conceptualization, and experimentation. There have been multiple instances in the literature of the effectiveness of an experiential learning model, including a 2025 study on social determinants of health where an experiential learning-based curriculum was developed that led to significant changes in student understanding of how social factors impact health [13]. In a 2018 study on experiential learning modules to teach integrative medicine, residents who underwent the hands-on modules demonstrated higher use of integrative medicine in their medical practice compared to residents who were exposed to the standard lecture model [14]. With this in mind, the procedure rotation was designed to incorporate supervised, hands-on experience with the medical team, supported by high-quality materials for self-directed learning and opportunities for formative feedback.

The procedure rotation included access to a self-directed online curriculum covering all of ABIM’s cognitive requirements (procedural indications, contraindications, and potential complications), standardized procedure guides based on peer-reviewed publications, open-access videos on procedures, and printable supply checklists. In addition to access to the online curriculum, students were given opportunities to observe and perform a variety of procedures, including paracentesis, thoracentesis, lumbar puncture, and knee arthrocentesis. Due to the on-demand nature of the rotation and the focus on real hands-on experience, simulation-based training was not incorporated in the curriculum. However, students had the opportunity to engage in self-directed simulation training in the open simulation lab throughout their pre-clinical and clinical years. To balance the procedural training of medical students with that of the residents on the procedure rotation, medical students generally performed procedures when the resident had been called in to cover another resident service or was otherwise unavailable. Medical students would also perform procedures if residents felt comfortable with their own experience and wanted to assist the medical student. If a medical student was not acting as the primary proceduralist, they would assist in consenting, set-up, and parts of the procedure itself. Consent was obtained by the residents or faculty with the help of the medical student. Patients were informed that the resident or the medical student would be performing the procedure under the supervision and guidance of faculty in the Division of Hospital Medicine. Patients were given the opportunity to ask questions and voice concerns. Students would start by observing and assisting in procedures performed by residents and faculty before they became primary operators. During and after procedures, students were coached in procedural technique, and feedback was provided in real time. Resident and student education was also supplemented with faculty-led procedure-specific didactics. Supervising faculty members were required to review the online curriculum and were internally trained on these procedures prior to supervising residents and students.

### Measured Outcomes

Students completed a knowledge-based assessment related to the cognitive aspects of procedures, including indications, contraindications, technical knowledge (such as landmarks), and interpretation of laboratory test results. The pre-elective cognitive assessment included 24 questions randomly selected from a pool of 54 questions, with an equal distribution of questions related to lumbar puncture, paracentesis, and thoracentesis. As the number of arthrocenteses was known to be low based on pre-intervention Procedure Service procedural volumes, arthrocentesis-specific cognitive assessment questions were not included. Students also completed a Likert (1–5) survey about procedural volume, confidence in performing procedures, and satisfaction with procedural training. The cognitive assessment and survey were administered before and after the rotation to measure change. The post-elective cognitive assessment was again a total of 24 questions randomly selected from the same pool of 54 questions. We assessed the impact of the intervention on patient safety by documenting any major complications of procedures performed by medical students. Major complications were defined as bleeding requiring transfusion or invasive intervention, bowel perforation, pneumothorax, spinal cord injury, or brain herniation.

### Statistical analysis

Statistical analysis was performed using R version 4.3.0. Cognitive assessment data are presented as individual raw scores as well as comprehensive mean scores and analyzed using a paired t-test. Questionnaire responses are presented as medians and stratified by procedure type. The number of procedure observations and attempts are presented as means and stratified by procedure type. Ordinal variables were compared using one-sided Wilcoxon matched-pairs signed-rank tests. A *p* value of < 0.05 was considered statistically significant for all analyses.

## Outcomes

**Fig. 1:**
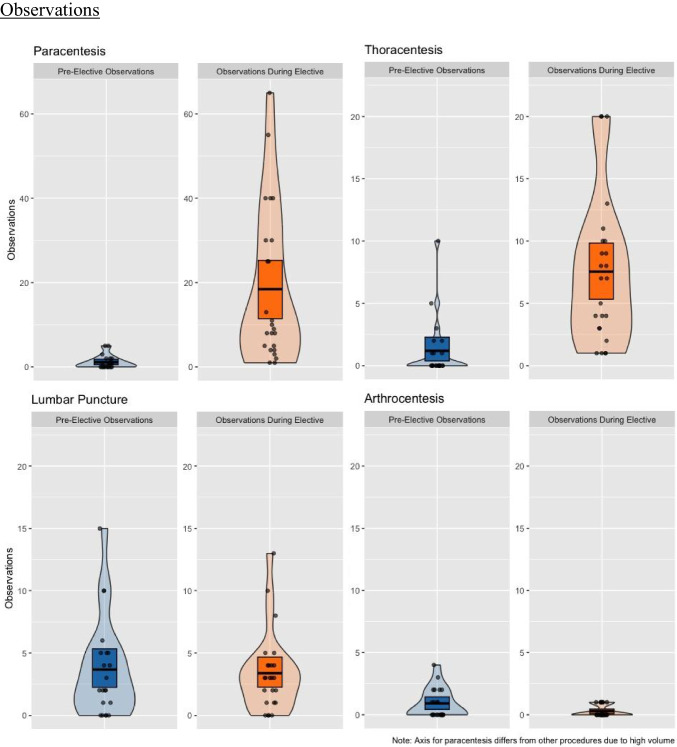

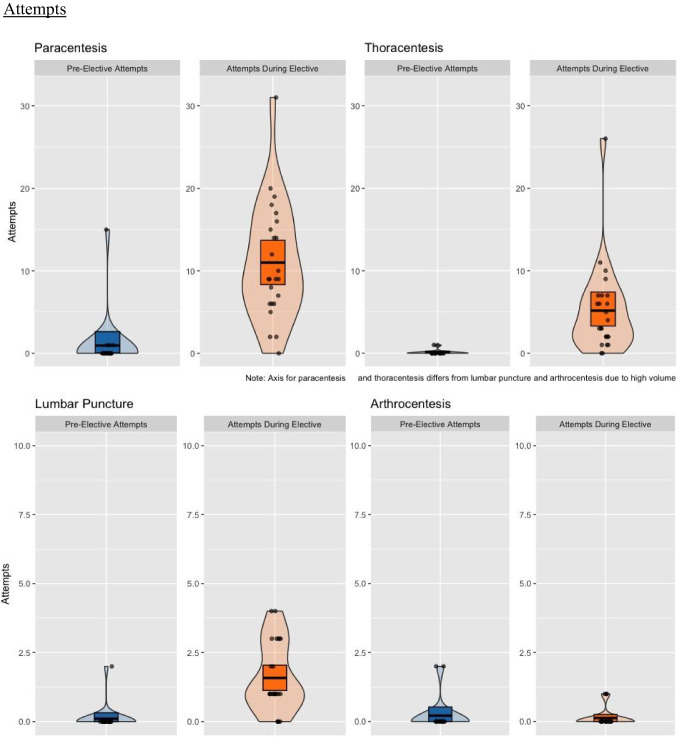
Mean student observed and attempted procedural volume pre- and post-implementation of the procedure rotation, separated by procedure

Twenty-four medical students participated in the intervention over a 3-year period. On average, students spent 6 weeks on the rotation. The pre-elective survey was completed by 87.5% of students, while the post-elective survey had a 100% response rate. Prior to the elective, the 24 students had performed a total of 26 procedures, with the majority reporting they had not performed any procedures. The total number of procedures performed by students participating in this rotation was 429. Medical students performed a mean of 11.0 paracenteses during the rotation (range 0.0–31.0), 5.2 thoracenteses (range 0.0–26.0), 1.6 lumbar punctures (range 0.0–4.0), and 0.12 arthrocenteses (range 0.0–1.0). These results are summarized in Fig. 1. There were zero major complications among the 429 procedures performed by students.

**Fig. 2:**
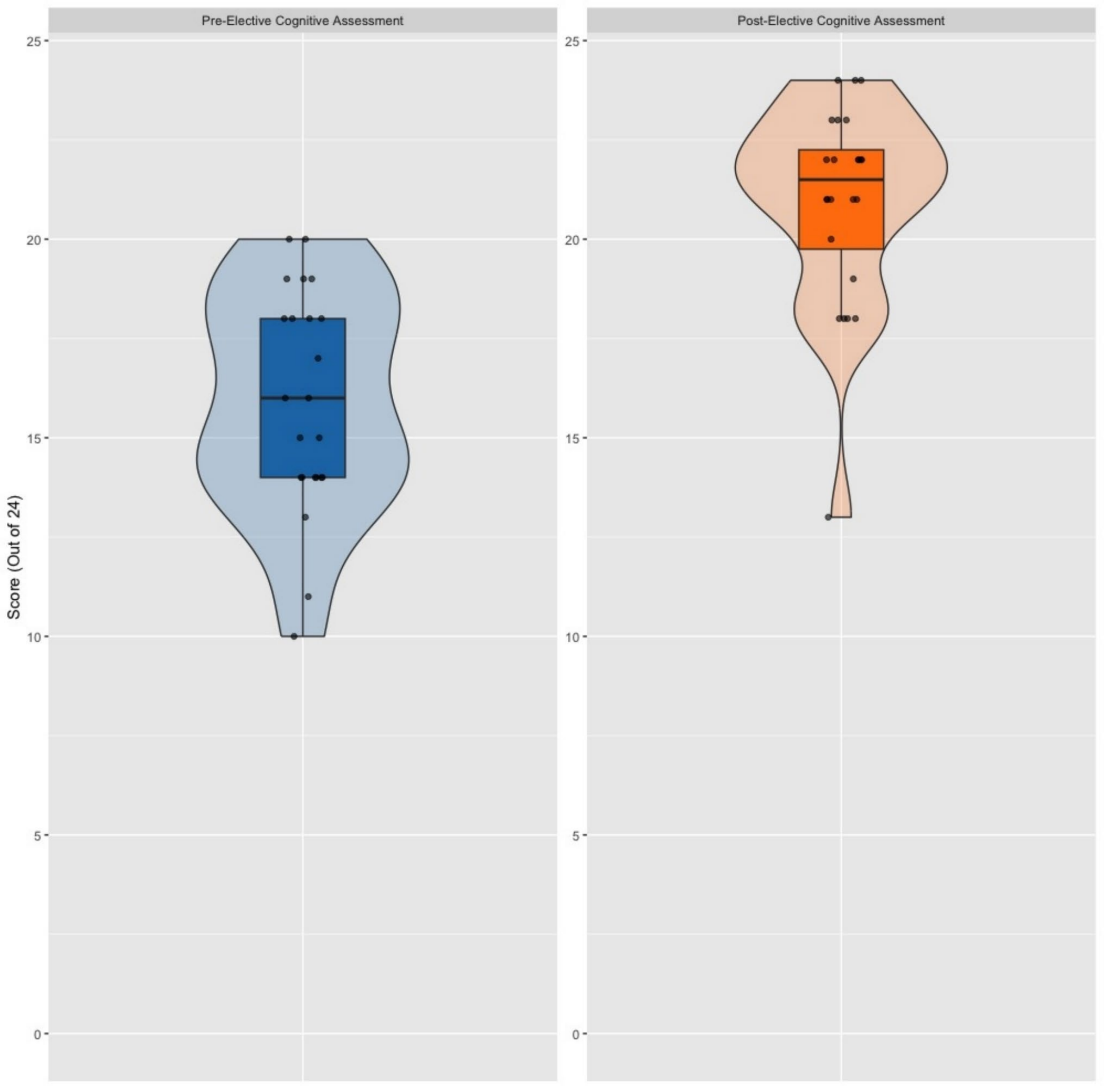
The cognitive assessment scores of participating students increased from a pre-intervention mean value of 66.3% to a post-intervention mean value of 87.2% (*p* < 0.001)

**Fig. 3:**
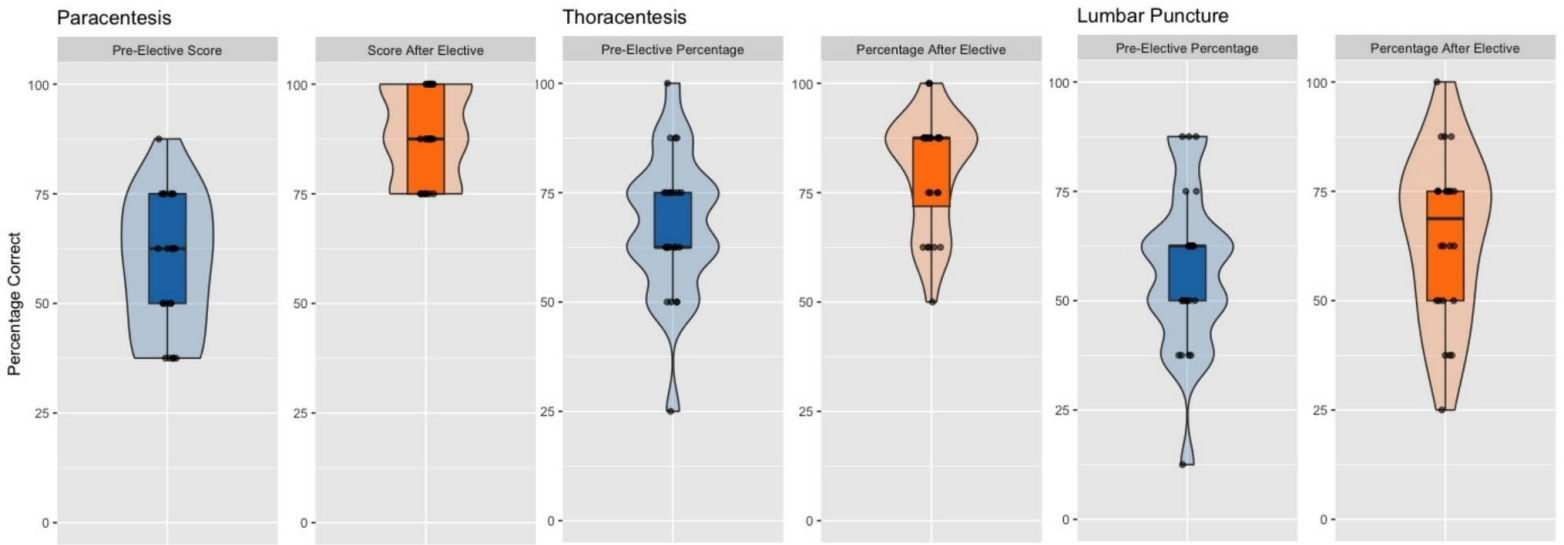
Stratified cognitive assessment scores based on procedure

Student scores on the knowledge-based quiz related to the cognitive aspects of internal medicine bedside procedures improved from a pre-intervention mean of 66.3% to a post-intervention mean of 87.2% (*p *< 0.001) (Fig. 2). The assessment included questions specific to paracentesis, thoracentesis, and lumbar puncture, allowing us to analyze changes in scores for each procedure separately. When the cognitive assessment results were stratified by procedure type—based on the specific questions related to paracentesis, thoracentesis, and lumbar puncture—improvement was seen for all three procedures. These improvements were statistically significant for the higher-volume procedures, paracentesis (*p* < 0.001) and thoracentesis (*p* = 0.005), while improvements for lumbar puncture (*p* = 0.111), the lower-volume procedure, were not significant (Fig. 3).

**Fig. 4:**
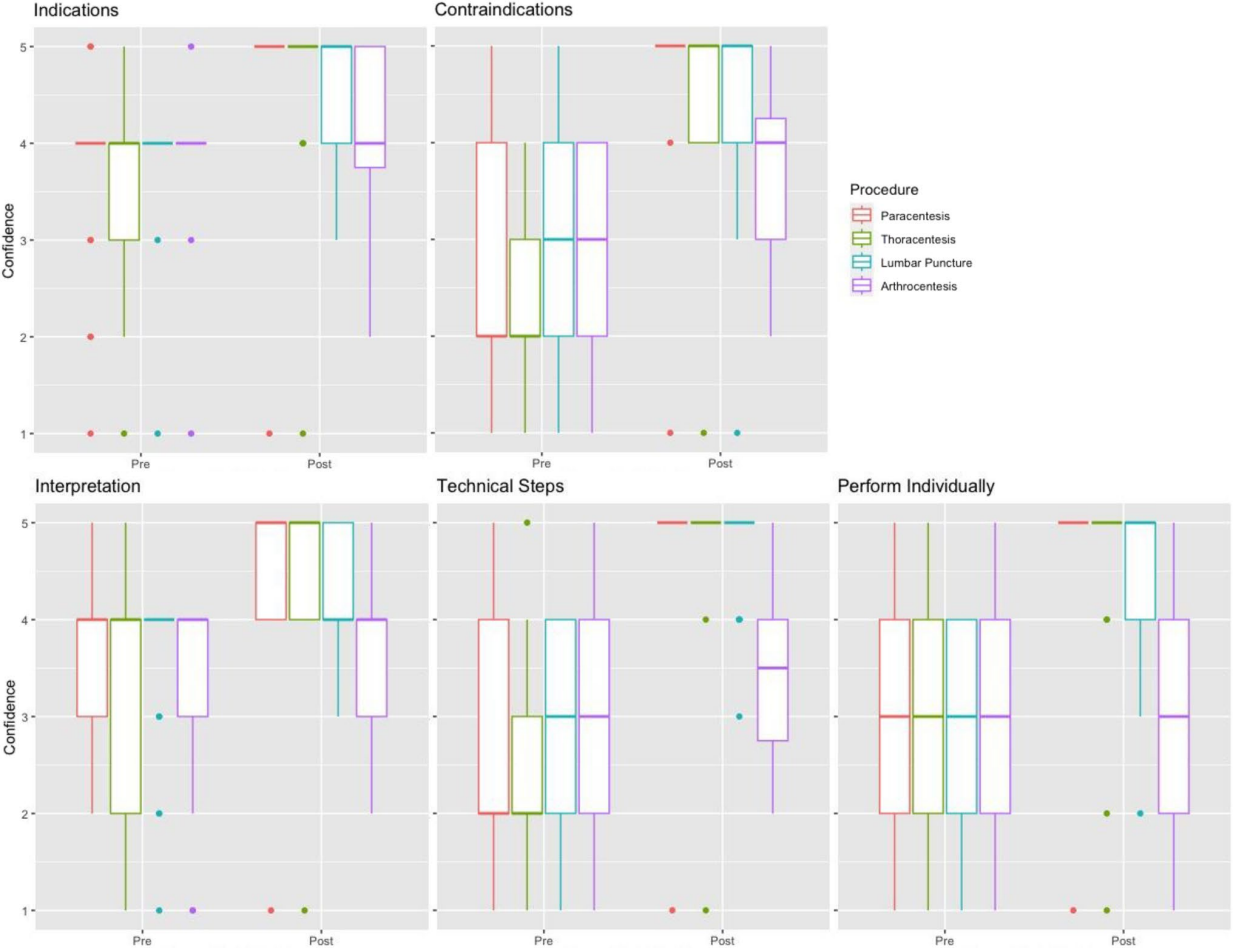
Pre- and post-rotation Likert questionnaire on procedural confidence and technical skills. Likert ratings indicate agreement with statements. Likert values ranging from 1 to 5: strongly disagree (1), disagree (2), undecided (3), agree (4), and strongly agree (5), respectively

Student confidence in performing paracentesis improved from a pre-intervention median of 3 to a post-intervention median of 5 (*p* < 0.001); for thoracentesis, from a pre-intervention median of 2.5 to a post-intervention median of 5 (*p *< 0.001); and for lumbar puncture, from a pre-intervention median of 3 to a post-intervention median of 5 (*p* = 0.001); there was no improvement in confidence performing knee arthrocentesis, with a pre-intervention median of 3 and a post-intervention median of 3 (*p* = 0.378). These results are summarized in Fig. 4. 

**Fig. 5:**
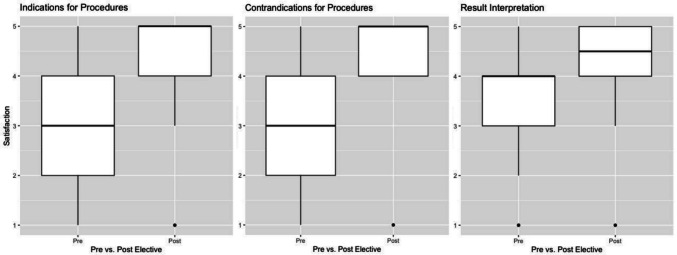
Pre- and post-rotation Likert questionnaire on satisfaction with procedural training. Likert ratings indicate agreement with statements. Likert values ranging from 1 to 5: strongly disagree (1), disagree (2), undecided (3), agree (4), and strongly agree (5), respectively

Student satisfaction with procedural training improved significantly across all measures (Fig. 5).

## Next Steps

We safely implemented an educational and experiential procedure rotation for medical students at a large academic medical center. Medical students were able to perform a number and variety of procedures that are generally not readily available at that stage in their training. While simulation training is well established in undergraduate and graduate medical education, direct clinical experience builds on prior knowledge, sharpens troubleshooting skills, strengthens bedside manner, and exposes learners to differences in patient body habitus, anatomical structures, and clinical scenarios. Additionally, students increased their understanding of the cognitive aspects of procedures and reported improvements in confidence in performing procedures and satisfaction with procedural training.

Students joined a preexisting procedure rotation that was established for residents. Prior to performing any procedures, students were provided with access to an online curriculum and were also participants in procedure-specific didactics led by faculty supervisors. Students typically began by observing procedures performed by residents and faculty and then gradually assisted with various steps of the procedure—such as selecting the procedure site and anesthetizing the skin—before eventually serving as the primary operator.

Of the 24 participating students, the majority (62%) reported that they had not performed a single paracentesis, thoracentesis, lumbar puncture, or arthrocentesis prior to the rotation. Compared to an average of 1 procedure prior to the elective, students performed an average of 18 procedures total during the rotation, the duration of which varied between 2 weeks, 4 weeks, and 8 weeks. One student performed as many as 31 paracenteses. The high volume of procedures students performed during the rotation likely contributed significantly to their reported increase in satisfaction with procedural training. Similarly, there was a statistically significant improvement in confidence performing the major bedside procedures featured in this rotation, namely paracentesis, thoracentesis, and lumbar puncture. Notably, there were few knee arthrocenteses performed by the Procedure Service and, indeed, an average of close to zero by participating students. As expected, there was no increase in student confidence performing knee arthrocentesis before or after the rotation, even with the inclusion of arthrocentesis didactics. This highlights the importance of active participation in improving procedural confidence.

There was a statistically significant increase in procedural knowledge following the intervention compared with baseline. Several factors likely led to this improvement. Students had access to an online curriculum that included procedure guides emphasizing cognitive elements, and within the experiential learning model, reviewing this didactic content before applying it in real procedures likely reinforced understanding. The dedicated nature of the rotation also allowed students ample time to engage with the material without competing classroom or clinical responsibilities. When procedural knowledge data were stratified by procedure, higher-volume procedures, paracentesis and thoracentesis, showed greater improvements in cognitive assessment scores compared to the lower-volume procedure, lumbar puncture. This pattern suggests that direct clinical experience enhances procedure-specific knowledge. This finding also helps distinguish the contributions of didactic materials from those of direct procedural experience. All students had access to the same online curriculum and procedure guides, yet knowledge gains were greatest for procedures with higher procedural volumes (paracentesis and thoracentesis) compared to the lower-volume lumbar puncture. The absence of a statistically significant improvement for lumbar puncture suggests that didactic exposure alone, without repeated real-patient experience, may be insufficient to produce comparable gains in cognitive performance. Arthrocentesis would have provided an even clearer example of a “didactic-only” procedure; however, it was excluded from the cognitive assessment due to its extremely low procedural volume during the study period, which limited its educational exposure and potential for meaningful statistical analysis.

Over time, the service has grown to an annual volume of approximately 400 procedures, as more surgical and medical services have relied on it for timely procedural support to advance patient care. This growth created enough procedural opportunities to accommodate additional learners, further enhancing the educational value of the service.

The experiential learning approach and the design of our procedure rotation is novel and can serve as a model for other academic medical centers. No similar experience for students has been described in the literature. One potential barrier to the implementation of such a rotation for students is the concern for patient safety. While studies have shown that resident-driven procedure services are associated with increased patient safety, no such data exist for services that include medical students.[6, 7, 9] We showed that students with minimal prior procedural experience can safely perform paracentesis, thoracentesis, and lumbar puncture in the setting of a standardized rotation led by a trained faculty supervisor. Another potential barrier is the ethical considerations around involving medical students in patient procedures. As mentioned previously, patients were consented and informed that a trainee (medical student or resident) would be performing the procedure under the direct supervision of trained faculty. Although we did not collect data on whether patients declined trainee involvement in their procedure, such occurrences were not commonly observed during the study period. This rotation provides an opportunity for students to engage in procedural training early in their medical career, allowing them to perform the same procedure multiple times under faculty guidance. Early exposure will help students develop important skills that they can continue to cultivate during their graduate medical education.

There are several limitations of this study. First, it was a single-center intervention with a relatively small sample size of students. In addition, several of the outcomes were self-reported, subjective, and based on self-assessment, using unvalidated tests and surveys. This study is also subject to selection bias, as students who selected the course were likely to be more interested in procedural medicine than their peers. Due to this sample size of self-selected learners, generalizability to other institutions and to other learners may be somewhat limited. Further, some academic centers may not have pre-existing procedure rotations for students to join, or sufficiently high procedure volume to accommodate additional learners. Finally, effort is required upfront to create an online curriculum featuring the procedure guides, links to standardized procedural videos, standardized procedural notes, and printable checklists.

This study describes the novel implementation of a procedure rotation for medical students at a large academic medical center. Student procedural volumes grew markedly as a result of the rotation, with associated improvement in procedural knowledge, confidence, and satisfaction with procedural training. This rotation was successfully implemented for multiple years, resulting in 429 total procedures performed by medical students with zero major complications. We believe this model is safe and well suited for academic medical centers in need of standardized and dedicated procedural training for medical students.

## Data Availability

The datasets generated and/or analyzed for this study are available from the corresponding author on reasonable request.
